# On the Metallic Basis of Alkalis

**Published:** 1809-01

**Authors:** 


### *To the Editors of the Medical and Physical Journal.*

Gentlemen,

In the days of Van Helmont and Becher, the first doubts originated, whether earths were simple bodies. By these chemists, an opinion was stated, that metals were capable of being produced from them. To this, however, little attention was paid, until the time of Lavoisier; in whose days day, and by whose principal aid, the world was enlightened by the discovery of oxygen. When it had been seen, that metals by combining with that body, lost all their characteristic properties, and assumed a pulverulent and earthy appearance, the conjecture elicited by Van Helmont and Becher was revived; and barytes being about this time discovered by Scheele, very material corroboration was afforded to the idea. Six or seven years afterwards, which is now about twenty years ago, Lavoisier published his Elements of Chemistry, where he developed the opinion at large. The idea coming from such authority, and derived from such plausible data, now gained considerable currency; and many of the most celebrated chemists of continental Europe directed their attention to the subject. Amongst these, Von Ruprecht, Tondi, and Baron Born, particularly distinguished themselves; and metallic buttons were believed to have been produced from the earths, in their experiments. To these conclusions, however, objections were urged by Klaproth and Savaresi, who stated, that the metals said to have been derived from the earths, were owing to impurities, which had been introduced with the materials employed; and that in fact, the buttons obtained, were phosphuret of iron. By some, Klaproth and Savaresi's statements were considered as decisive; while others looked upon them as not carrying considerable weight: and now that we find the earths are oxydes of metals, some ground is afforded for giving a certain degree of credit to this latter opinion.

Hitherto we have seen the earths *only* had been suspected to be of a compound nature. That idea was never extended to the alkalis, until some years afterwards. The reasons that existed for cherishing a suspicion of the sort, with regard to the latter class of bodies, were the alkaline nature of barytes, magnesia, and lime; and although these were taken no notice of by the foreign chemists, they did not escape the observation of an acute countryman of our own, Mr. Kerr, a short time afterwards. This gentleman was the translator of Lavoisier's Elements, and to that part of the work, where Lavoisier states his conjectures with regard to the earths, Mr. Kerr added an account of the experiments of Von Ruprecht, Tondi, and Born. This he accompanied with comments; and seizing the idea of the alkaline nature of four of the earths (for strontian had, at this date, been added by Dr. Hope) he developed from a fine train of reasoning, the opinion that "all bodies in nature may be divided into oxygen, inflammable matter, and caloric, caloric,"* that "*alkalinity* and acidity depended upon the doses of oxygen with which the combustible was combined"†; and, in fine, that "*the alkalies were* metallic substances *in some hitherto unknown state of combination*"‡.

These predictions have within the last few months been realized; and in realizing them, Davy has rendered himself immortal. But hitherto, the tide of admiration has carried us so swiftly in its current, that we have not been able to look back with sufficient steadiness upon the objects by which it has been excited. Now, that the wave is on the ebb, however, we have leisure to cast a more enquiring eye around us, and to apportion to each, the share of praise which his exertions have demanded. To Mr. Davy, then, we give the first and greatest, for having *completed* so great an æra in the science. In Volta we have a most deserving claimant, for our next; since to him we are indebted for the instruments by which the task has been accomplished. And in turning our eyes towards Lavoisier and Kerr, we find two meritorious philosophers, on whom we have to bestow, by no means an inferior share of admiration; for from them did we receive the spark, which, by the application of means derived from Volta, the fostering genius of Davy has kindled into flame. To Lavoisier and Kerr, then, for the outline—to Volta for the materials—and to Davy for the construction of the fabric, be our praises respectively directed.

I am, &c.

JACOBUS.

* Lavoisier's Elements of Chemistry, 5th Edit. Vol. I, p. 265.

